# *Legionella pneumophila* Carbonic Anhydrases: Underexplored Antibacterial Drug Targets

**DOI:** 10.3390/pathogens5020044

**Published:** 2016-06-16

**Authors:** Claudiu T. Supuran

**Affiliations:** Dipartimento Neurofarba, Sezione di Scienze Farmaceutiche e Nutraceutiche, Università degli Studi di Firenze, Via U. Schiff 6, Sesto Fiorentino, Florence 50019, Italy; claudiu.supuran@unifi.it; Tel./Fax: +39-055-4573729

**Keywords:** *Legionella pneumophila*, carbonic anhydrase, bacteria, inhibitor, antibiotic, virulence factor, sulfonamide

## Abstract

Carbonic anhydrases (CAs, EC 4.2.1.1) are metalloenzymes which catalyze the hydration of carbon dioxide to bicarbonate and protons. Many pathogenic bacteria encode such enzymes belonging to the α-, β-, and/or γ-CA families. In the last decade, enzymes from some of these pathogens, including *Legionella pneumophila*, have been cloned and characterized in detail. These enzymes were shown to be efficient catalysts for CO_2_ hydration, with k_cat_ values in the range of (3.4–8.3) × 10^5^ s^−1^ and k_cat_/K_M_ values of (4.7–8.5) × 10^7^ M^−1^·s^−1^. *In vitro* inhibition studies with various classes of inhibitors, such as anions, sulfonamides and sulfamates, were also reported for the two β-CAs from this pathogen, LpCA1 and LpCA2. Inorganic anions were millimolar inhibitors, whereas diethyldithiocarbamate, sulfamate, sulfamide, phenylboronic acid, and phenylarsonic acid were micromolar ones. The best LpCA1 inhibitors were aminobenzolamide and structurally similar sulfonylated aromatic sulfonamides, as well as acetazolamide and ethoxzolamide (K_I_s in the range of 40.3–90.5 nM). The best LpCA2 inhibitors belonged to the same class of sulfonylated sulfonamides, together with acetazolamide, methazolamide, and dichlorophenamide (K_I_s in the range of 25.2–88.5 nM). Considering such preliminary results, the two bacterial CAs from this pathogen represent promising yet underexplored targets for obtaining antibacterials devoid of the resistance problems common to most of the clinically used antibiotics, but further studies are needed to validate them *in vivo* as drug targets.

## 1. Introduction

Resistance to antibiotics belonging to all pharmacological classes is escalating and represents a worldwide problem [1,2,3,4,5,6,7,8,9,10]. Both Gram-negative and Gram-positive bacteria (such as *Staphylococcus aureus*, *Mycobacterium tuberculosis*, *Helicobacter pylori*, *Brucella suis*, *Streptococcus pneumoniae*, *etc.*) no longer respond to many clinically used such drugs belonging to several antibiotic classes [9,10,11,12]. Cloning of the genomes of many bacterial pathogens offers the possibility to explore alternative pathways for inhibiting virulence factors or proteins essential for their life cycles [8,13,14,15,16,17]. Among many such new possible drug targets explored recently, there is a class of enzymes catalyzing a simple but physiologically relevant process, *i.e.*, the hydration carbon dioxide to bicarbonate and protons, generating thus a buffering weak base (bicarbonate) and a strong acid (H^+^) [18,19,20,21,22,23]. These metalloenzymes are the carbonic anhydrases (CAs, EC 4.2.1.1). Six genetically distinct CA families are known to date, the α-, β-, γ-, δ-, ζ- and η-CAs [18,24,25,26,27,28,29]. The α-, β-, δ- and η-CAs use Zn(II) ions at the active site, the γ-CAs are probably Fe(II) enzymes (but they are also active with bound Zn(II) or Co(II) ions), whereas the ζ-class uses Cd(II) or Zn(II) to perform the physiologic reaction catalysis [18,22,26,27].

The 3D fold of these enzyme classes are very different from each other (a nice example of convergent/divergent evolution [22]), as it is their oligomerization state: α-CAs are normally monomers and rarely dimers; β-CAs are dimers, tetramers, or octamers; γ-CAs are trimers [28], whereas the δ-, ζ- and η-CAs are probably monomers but in the case of the ζ-CA family, three slightly different active sites are present on the same protein backbone (which is thus a pseudotrimer, at least for the best investigated such enzyme, from the diatom *Thalassiosira weissflogii*) [29]. Many representatives of all these enzyme classes have been crystallized and characterized in detail, except for representatives of the δ- and η-CAs, for which the exact structure is not known to date [22]. The mammalian CAs and their inhibitors/activators have been thoroughly reviewed elsewhere [22,23,24] and these enzymes will be not discussed here, although they are off-targets when considering bacterial CAs as possible anti-infective drug targets [5,8].

These enzymes are ubiquitous: the α-CAs are present in vertebrates, protozoa, algae, and cytoplasm of green plants and in some *Bacteria*; the β-CAs are predominantly found in *Bacteria*, algae and chloroplasts of both mono- as well as dicotyledons, but also in many fungi and some *Archaea* [28,29,30,31,32]. In bacteria and fungi they are mostly homodimers/tetramers [30]. The γ-CAs were found in *Archaea* and *Bacteria*, whereas the δ- and ζ-CAs seem to be present only in marine diatoms [28,29,30,31,32]. In most organisms, these enzymes are involved in crucial physiological processes connected with respiration and transport of CO_2_/bicarbonate, pH and CO_2_ homeostasis, electrolyte secretion in a variety of tissues/organs, biosynthetic reactions (such as gluconeogenesis, lipogenesis and ureagenesis), bone resorption, calcification, tumorigenicity, and many other physiologic or pathologic processes (thoroughly studied in vertebrates), whereas in algae, plants, and some bacteria they play an important role in photosynthesis and biosynthetic reactions. In diatoms δ- and ζ-CAs play a crucial role in carbon dioxide fixation [28,29].

Bacteria are ubiquitous microorganisms, with most available niches occupied by many members of this kingdom. Bacteria encode CAs from three classes, the α-, β-, and γ-CA class, thus suggesting that these enzymes play an important role in the prokaryotic physiology [5,8]. It has been demonstrated that these three families are genetically quite distinct between each other [5]. Furthermore, the various members of the same class from diverse organisms possess very different biochemical properties, 3D folds of the protein backbone (for those enzymes for which the structure has been resolved by means of X-ray crystallography [5,20,22]), and susceptibility to be inhibited by various classes of CA inhibitors (CAIs), such as the sulfonamides and the inorganic anions, the most investigated such modulators of activity [8,33,34,35].

*Legionella pneumophila* is a Gram-negative bacterium causing Legionnaires’ disease or legionellosis, an often fatal pneumonia (mortality rate of 20%–50%), which was observed for the first time in July 1976, among the attendees of the 58th Annual Convention of the American Legion in Philadelphia [36,37,38,39]. There are many species of this bacterium, but only *L. pneumophila* and to a lower extent *L. longbeachae* provoke disease in humans (the last species is prevalent in Australia and New Zealand, whereas the first one in the other continents) [36]. *Legionellae* are environmental bacteria and their natural host is the amoebae in which they replicate, but by accidentally infecting human macrophages they cause oportunistic infections [40,41]. The spread of legionellosis was favored ultimately by the development of artificial water systems for air conditioning, cooling towers, aerosolizing devices, *etc.* [40].

The mechanisms by which these bacteria infect their hosts include the formation of *Legionella*-containing vacuoles (LCVs) within the host cytoplasm, which requires the remodeling of the LCV surface and the hijacking of vesicles and organelles for effective infection [39]. These processes are mainly controlled by the pH of these organelles, and as a consequence the pathogen, similar to other bacteria, fungi, or protozoa [5,8,18,19,20], evolved mechanisms of interfering with the pH. Both macrophages and amoebae use phagosomes to destroy foreign particles/pathogens, in which a highly acidic pH is crucial for the outcome of the process [40]. Intracellular pathogens, such as *L. pneumophila* thus developed sophisticated strategies to evade their destruction by the acidic phagolysosomes [40,41]. Indeed, it has been demonstrated that *L. pneumophila* is able to maintain a neutral pH in its phagosome for at least 6 h, whereas vacuoles which did not contain the bacterium became highly acidic in 15 min after their formation [42]. One of the proteins involved in this process is a vacuolar V-ATPase [40], but as for other organisms [5,8,18,19,20], the pH regulation is a complex process in which many other proteins are involved, among which the CAs (which generate protons and bicarbonate by the hydration of the highly available substrate CO_2_) [43,44]. Thus, the investigation of *L. pneumophila* CAs started to interest researchers due to the potential of such proteins to develop alternative antibiotics [43,44]. Here I shall review the developments in the field of the cloning, characterization, and inhibition of the two such enzymes from this pathogen.

## 2. Cloning and Biochemical Properties of LpCA1 and LpCA2, the β-CAs from *L. pneumophila*

The genome of *Legionella pneumophilia* subsp. *pneumophila* strain Philadelphia-1 has been cloned some years ago [41], and inspection of the genome revealed the presence of two putative such enzymes belonging to the β-class, denominated LpCA1 and LpCA2 [43,44]. They have been cloned as glutathione *S*-transferase (GST)-fusion proteins [43,44]. LpCA1 contains 245 amino acid residues and has a calculated molecular weight of 26.994 kDa, whereas LpCA2 is shorter, comprising 208 amino acid residues, with a theoretical molecular weight of 22.997 kDa.

**Figure 1:**
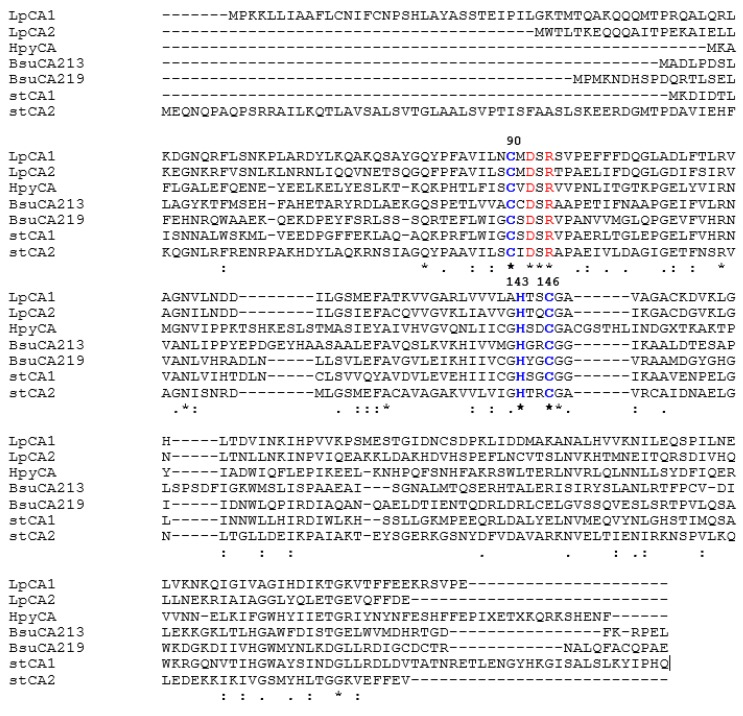
Amino acid sequence alignment of selected β-CAs from four bacterial species. LpCA1 numbering system was used. Amino acid residues participating in the coordination of metal ion are indicated in blue, whereas the catalytic dyad involved in the activation of the metal ion coordinated water molecule (Asp92–Arg94) is shown in red. The asterisk (*) indicates identity at a position; the symbol (:) designates conserved substitutions, whereas (.) indicates semi-conserved substitutions. The multiple alignment was performed with the program MUSCLE and refined using the program Gblocks. Organisms, NCBI sequence numbers, and cryptonyms are indicated in Table 2.

We have aligned the amino acid sequences of LpCA1 and LpCA2 with those of other such enzymes characterized earlier (kinetically, and in some cases also by means of X-ray crystallography), such as HpyCA from *Helicobacter pylori* [45], BsuCA213 and BsuCA219 (from *Brucella suis* [46,47]) and stCA1 and stCA2 from the bacterial pathogen *Salmonella typhimurium* [48,49] (Figure 1). Data of Figure 1 show that, as all other bacterial β-CAs investigated so far, the two *Legionella* proteins LpCA1 and LpCA2 possess the amino acid residues crucial in the catalytic cycle of CO_2_ conversion to bicarbonate and protons: (i) the zinc coordinating amino acids, constituted by one His and two Cys residues, more precisely Cys90, His143, and Cys146 (LpCA1 numbering system); and (ii) the catalytic dyad constituted by residues Asp92 and Arg94, which is involved in the activation of the zinc-coordinated water molecule which leads to the formation of the nucleophilic, zinc-hydroxide species of the enzyme [43,44].

The measured CO_2_ hydrase activity of the two enzymes, LpCA1 and LpCA2 (Table 1), where the activity of LpCA1 and LpCA2 was compared to that of other α- and β-CAs from human (h), fungal (*Cryptococcus* *neoformans* [50], *Candida albicans* [51], *Saccharomyces cerevisiae* [52]), and bacterial (*H. pylori* [45] and *B. suis* [46,47]) sources, showed that both these enzymes possess a significant catalytic activity for the physiologic reaction catalyzed by these enzymes, *i.e.*, hydration of carbon dioxide with formation of protons and bicarbonate. The first isoform, LpCA1 showed a moderate degree of activity with a k_cat_ of 3.4 × 10^5^ s^−1^ and k_cat_/K_m_ of 4.7 × 10^7^ M^−1^·s^−1^. This activity is in fact comparable to that of other α- and β-CAs, such as hCA I (a physiologically relevant human isoform) [18], Can2 from the fungal pathogen *C. neoformans* [50], and the bacterial pathogenic enzymes HpyCA and BsuCA219, from *H. pylori* and *B. suis*, respectively [45,46]. The second isoform, LpCA2, was on the other hand more active than LpCA1, with the folllowing kinetic parameters: k_cat_ of 8.3 × 10^5^ s^−1^ and k_cat_/K_m_ of 8.5 × 10^7^ M^−1^·s^−1^ (Table 1). This is a level of activity similar to that of the *C. albicans* and *S. cerevisiae* enzymes or the BsuCA213 isoform (all belonging to the β-CA family) and is only slightly lower compared to hCA II, a highly effective catalyst [18] for the CO_2_ hydration reaction (Table 1). It may also be observed that acetazolamide (5-acetamido-1,3,4-thiadiazole-2-sulfonamide) **AAZ**, a clinically used drug, inhibited efficiently the two *Legionella* enzymes, with K_I_s in the range of 36–95 nM. The moderately active enzyme (LpCA1) was less sensitive to this compound compared to LpCA2 which showed a higher catalytic activity and also higher affinity for the sulfonamide inhibitor (Table 1).

**Figure 2:**
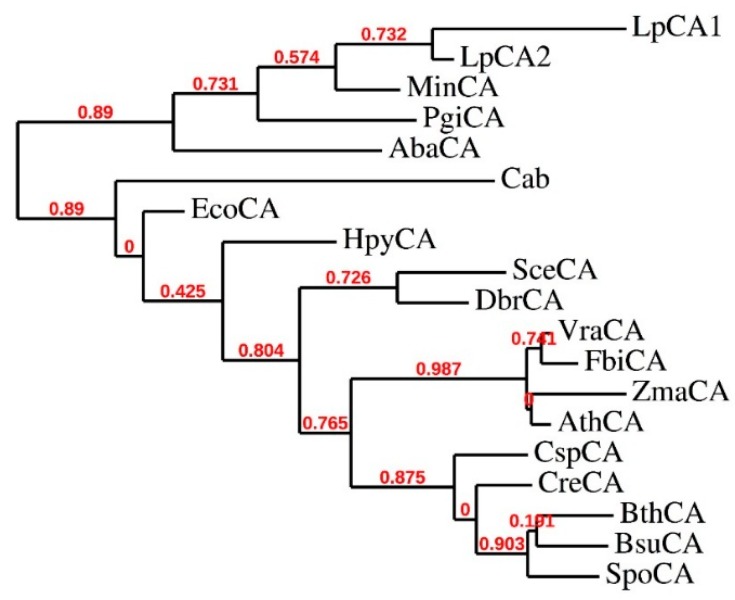
Phylogenetic analysis of β-CA amino acid sequences of organisms shown in Table 2. The tree was constructed using the program PhyML 3.0, phylogeny software based on the maximum-likelihood principle [44]. Branch support values are reported at each branch point.

We have also examined the phylogenetic relationship of the two new bacterial β-CAs, LpCA1 and LpCA2, with that of other such enzymes characterized earlier from various bacterial and fungal pathogens, as well as algae, plants, and Archaea (see Table 2 for the organisms included in the phylogenetic analysis). As seen from the dendrogram of Figure 2, the two *Legionella* enzymes are highly similar, clustering on their own branch at the upper part of the tree, with the next most similar enzyme being MinCA from the recently identified bacterial pathogen resistant to all known antibiotics *Myroides* *injenesis* (this β-CA was not yet cloned and characterized) [44]. On the other hand, the other evolutionarily most related enzyme to LpCA1 and LpCA2 is AbaCA, from *Acinetobacter baumanii*, another not yet cloned or characterized β-CA. The remaining β-CAs from Figure 2 were less related to LpCA1 and LpCA2 compared to the previously discussed ones, and clustered all together on the lower branches of the tree. This includes two main branches, one constituted only by the archaeal enzyme cab (from *Methanobacterium thermoautotrophicum*) [53], whereas the remaining, rather ramificated, branch includes yeast/fungal (SceCA, DbrCA and SpoCA), bacterial (HpyCA, BsuCA), algal (CspCA and CreCA), and plant enzymes (VraCA, FbiCA, ZmaCA and AthCA), which are all more distantly related to the bacterial CAs from *Legionella* examined here (Figure 2).

## 3. Sulfonamide/Sulfamate Inhibition Studies of LpCA1 and LpCA2

**Chart 1:**
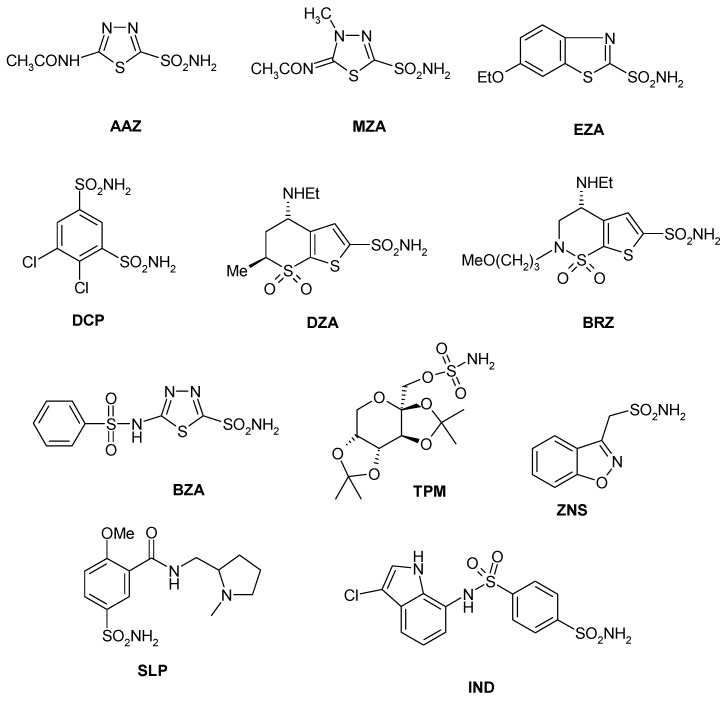

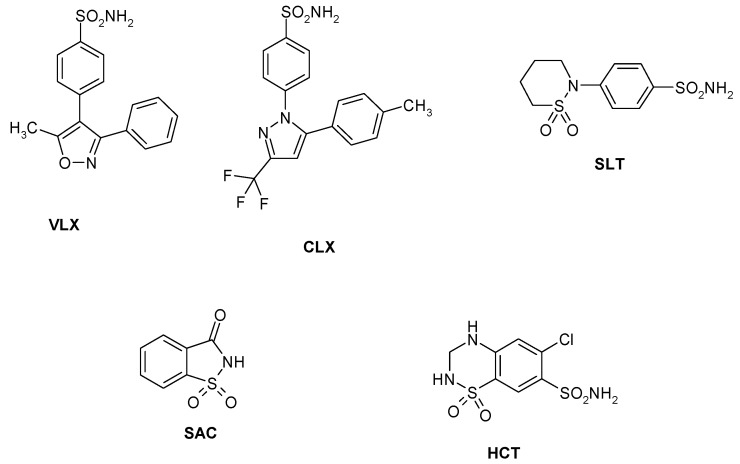
Sulfonamide/sulfamate CAIs investigated as LpCA1 and LpCA2 inhibitors.

Sulfonamides and their isosteres (sulfamates, sulfamides, *etc.*) constitute the main and most investigated family of CAIs, with several drugs used clinically for the last 60 years [18]. Among them are first generation compounds such as acetazolamide **AAZ**, methazolamide **MZA**, ethoxzolamide **EZA** and dichlorophenamide **DCP**. Dorzolamide **DZA** and brinzolamide **BRZ** are topically-acting antiglaucoma agents belonging to the second generation of such drugs [54], whereas benzolamide **BZA** is an orphan drug belonging to this class of pharmacological agents [18,19,20,21,22]. Topiramate **TPM** (a sulfamate) and zonisamide **ZNS** (an aliphatic sulfonamide) are widely used antiepileptic drugs [55]. Other compounds incorporating primary/secondary sulfamoyl moieties such as sulpiride **SLP** and indisulam **IND** were also shown to belong to this class of pharmacological agents together with the COX2 selective inhibitors celecoxib **CLX** and valdecoxib **VLX**, the antiepileptic sulthiame **SLT**, the sweetener saccharin **SAC**, and hydrochlorothiazide **HCT**, a diuretic agent [56]—see Chart 1. Compound **1**–**24** are sulfonamides possessing a variety of scaffolds/substitution patterns, which were included in the study [44] in order to explore a wider chemical space for detecting effective inhibitors of these CAs, (Table 3).

Data of Table 3 show the inhibition of the two *Legionella* enzymes, LpCA1 and LpCA2 with this set of sulfonamides/sulfamates. For comparison reasons, the inhibition of the two human (h) offtarget isoforms hCA I and II (belonging to the α-CA family) as well as the bacterial β-CA from the pathogen *H. pylori* (HpyCA) with this set of 40 compounds, are also presented in Table 3. The following structure-activity relationships (SAR) were observed for the inhibition of LpCA1 and LpCA2 with these compounds:

(i) In the case of the slow isoform LpCA1, the less effective inhibitors were **SAC** and **HCT** which showed affinities in the micromolar range (K_I_s of 15.8–20.5 µM), whereas compounds **1**–**3**, **5**–**11**, **14**–**18**, **DCP**, **DZA**, **ZNS**, **IND,** and **CLX** were poorly effective as CAIs against LpCA1 (K_I_s in the range of 734–3540 nM, Table 3). Benzenesulfonamides with a more complicated substitution pattern (**DCP**, **IND,** or **CLX**) were also weak—medium potency inhibitors of this enzyme, together with heterocyclic sulfonamides such as **14**, **DZA**, and **ZNS**.
(ii) A better inhibitory power against LpCA1, with inhibition constants ranging between 101 and 665 nM was observed for the following derivatives: **12**, **13**, **19**, **21**, **22**, **MZA**, **BRZ**, **BZA**, **TPM**, **SLP**, **VLX**, and **SLT**. An increase of LpCA1 inhibitory activity was seen for **12** (compared to the structurally related **11**) or **13** over the structurally related **14**. In fact the two pairs of compounds are rather similar: **12** has a chlorine instead the CF_3_ moiety of **11**, but their K_I_s differ by a factor of 1.44. Thus, rather small structural changes in the scaffold of the inhibitors lead to important changes in the inhibitory power of the compound against the LpCA1 enzyme.
(iii) The best LpCA1 inhibitors were **20**, **23**, **24**, **AAZ**, and **EZA** (K_I_s in the range of 40.3–90.5 nM, Table 3). In this case the SAR is very interesting. In addition to the heterocyclic derivatives **AAZ** and **EZA**, which are usually highly potent CAIs of most investigated Cas [18], acting thus as promiscuous inhibitors, the potent LpCA1 inhibitors possessed a rather similar structure of elongated, sulfonylated sulfonamide type. Aminobenzolamide **20** (and benzolamide **BZA**) are the prototype of such compounds, but interestingly, the aromatic compounds **23** and **24** were the best LpCA1 inhibitors. Furthermore, LpCA1 inhibition clearly increased with an increase in the spacer between the two aminobenzenesulfonyl fragments from the molecules. Indeed, **23** and **24** showed K_I_s of 59.8 and 40.3 nM, respectively (Table 3).
(iv) The fast *Legionella* isoform, LpCA2 was also inhibited by all sulfonamides/sulfamates investigated so far (Table 3). The inhibition range was however not as wide as for the previous isoform (LpCA1), as the best LpCA2 inhibitor showed a K_I_ of 25.2 nM and the worst one of 933 nM. A small number of the investigated sulfonamides/sulfamates were quite weak LpCA2 inhibitors, among which compound **3**, **TPM**, **ZNS**, **VLX,** and **HCT** (K_I_s in the range of 745–933 nM, Table 3).
(v) Many of the investigated sulfonamides were medium potency LpCA2 inhibitors, with K_I_s in the range of 103–721 nM. They include: **1**, **2**, **4**–**19**, **EZA**, **DZA**, **BRZ**, **BZA**, **SLP**, **IND**, **CLX**, **SLT**, and **SAC** (Chart 1 and Table 3). Simple benzenesulfonamide incorporating one or two substituents of the amino, aminoalkyl, hydroxy, hydroxyalkyl, halogens, sufamoyl *etc.* type, were not very different in their behavior as LpCA2 inhibitors, all of them leading to the medium potency inhibition profiles. The various aromatic/heterocyclic scaffolds present in the clinically used drugs **EZA**, **DZA**, **BRZ**, **BZA**, **SLP**, **IND**, **CLX**, **SLT**, and **SAC** were also comparable to the simple scaffolds present in compounds **1**, **2,** and **3**–**19**.
(vi) The best LpCA2 inhibitors were **20**–**24**, **AAZ**, **MZA,** and **DCP** (K_I_s in the range of 25.2–88.5 nM, Table 3). SAR is highly interesting here. Except the clinically used drugs (**AAZ**, **MZA,** and **DCP**) which have not much in common, all the other effective LpCA2 inhibitors possess the same scaffold, of the arylsulfonylated aminosulfonamide type. Thus, aminobenzolamide **20** was 3.3 times more effective as LpCA2 inhibitor compared to benzolamide **BZA**, whereas for the aromatic componds **22**–**24**, as for LpCA1, the activity increases with the increase of the molecule length, the best LpCA2 inhibitor being **24** (this was also the best LpCA1 inhibitor detected so far).
(vii) Except for some of the effective CAIs detected here, which showed a good activity against both LpCA1 and LpCA2 (e.g., **22**–**24** and **AAZ**), generally the two isoforms had a rather different affinity for these inhibitors. For example **SAC** was a very weak LpCA1 inhibitor but a medium potency LpCA2 inhibitor. The same behavior was observed for **DCP**. Most of the time, these compounds showed an enhanced inhibition of LpCA2 over LpCA1, although several compounds with the reverse profile (e.g., **4**, **EZA**, **VLX**) were also detected.
(viii) The inhibition profiles of the two *Legionella* enzymes is very different compared to that of other bacterial β-CAs (e.g., HypCA) or the off-target, human isoforms hCA I and II (Table 3). This is of interest in case some of these compounds should be used for targeting the bacterial over the human isofoms in experimental or clinical settings.

## 4. Inorganic Anions and Other Small Molecule LpCA1/LpCA2 Inhibitors

Anions and other small molecules (e.g., sulfamide, sulfamate, phenylboronic acid, or phenylarsonic acid) represent a class of well-established CAIs, as they bind to the metal ion from the enzyme active site and impair catalysis [18,34,35]. An inhibition study of the two *Legionella* enzymes with a range of such inhibitors, including simple and complex inorganic anions as well as sulfamide, sulfamate, phenylboronic acid, and phenylarsonic acid was thus reported [43] (Table 4). The following has been noted regarding inhibition of the two enzymes by the anions and small molecules shown in Table 4.

(i) Perchlorate and tetrafluoroborate did not inhibit the two new β-CAs reported here (K_I_ > 200 mM). Similar results have been observed in most of the CAs examined to date: only HpyCA was effectively inhibited by perchlorate, with a K_I_ of 6.5 mM [45]. Sulfate was also an ineffective LpCA1/LpCA2 inhibitor, with K_I_ values between 77.9–96.5 mM (Table 4). Iodide and nitrate were also quite weak LpCA2 inhibitors, with inhibition constants of 59.1 and 30.1 mM, respectively.
(ii) Another group of anions inhibited LpCA1 and LpCA2 weakly, with inhibition constants in the range of 3.5–9.1 mM. They include bicarbonate, carbonate, nitrate, nitrite, hydrogen sulfite, selenate and fluorosulfonate against LpCA1, whereas for LpCA2, the weak inhibitors included bromide, bicarbonate, carbonate, nitrite, and hydrogen sulfite (Table 4).
(iii) A large number of the anions acted as submillimolar inhibitors against both these enzymes. All of the halides, cyanate, thiocyanate, stannate, tellurate, pyrophosphate, divanadate, tetraborate, perrhenate, perruthenate, peroxydisulfate, selenocyanate, and trithiocarbonate inhibited LpCA1 with K_I_ values from 0.24–0.98 mM. Iminodisulfonate was less effective as an LpCA1 inhibitor (K_I_ of 1.17 mM). The effective, submillimolar LpCA2 inhibitors were fluoride, chloride, cyanate, thiocyanate, cyanide, azide, hydrogen sulfide, stannate, tellurate, pyrophosphate, divanadate, tetraborate, perrhenate, perruthenate, peroxydisulfate, selenocyanate, trithiocarbonate, fluorosulfonate, and iminodisulfonate (K_I_ values ranging from 0.29–0.96 mM, Table 4). The best inhibitor in this subseries was tellurate, with K_I_ values of 0.24 and 0.29 mM against LpCA1 and LpCA2, respectively.
(iv) The best anionic LpCA1 inhibitors were cyanide, azide, hydrogen sulfide, diethyldithiocarbamate, sulfamide, sulfamate, phenylboronic acid, and phenylarsonic acid (K_I_ of 6–94 µM). *N*,*N*-diethyldithiocarbamate had a much higher affinity for LpCA1, with a low micromolar value for K_I_ of 6 µM. However, all of the small molecules were low micromolar inhibitors of LpCA2, with K_I_ values ranging between 2 and 13 µM (Table 4).
(v) There are net differences in the behavior of the two *Legionella* enzymes towards the anionic inhibitors investigated here. LpCA1 showed higher affinity for some poisonous metal anions such as cyanide, azide, and hydrogen sulfide, which are around one order of magnitude more potent inhibitors against LpCA1 than LpCA2. However, LpCA2 showed higher affinity for *N*,*N*-diethyldithiocarbamate, sulfamide, sulfamate, phenylboronic acid, and phenylarsonic acid compared to LpCA1.

**Figure 3:**
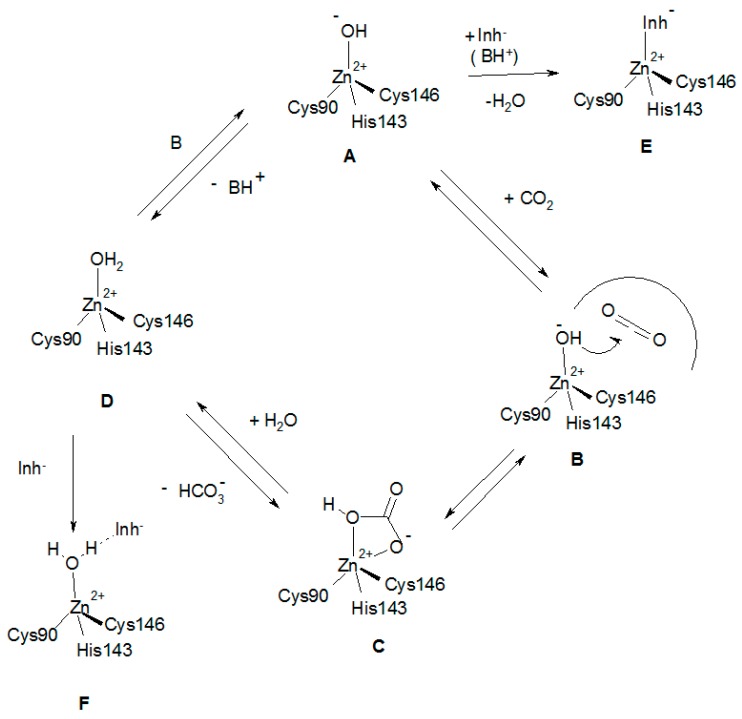
Proposed catalytic/inhibition mechanisms of LpCAs (exemplified by using LpCA1, and its amino acid residues numbering system) [43]. The catalytic cycle for the CO_2_ hydration is shown in the clockwise direction, the bicarbonate dehydration in the counterclockwise one. See text for details.

A tentative catalytic/inhibition mechanism of the two LpCA1/2 enzymes is presented in Figure 3, based on crystallographic and kinetic data published for similar β-CAs [5,8,14,15,23,30], as the X-ray crystal structures of the two *Legionella* enzymes is not known at the moment.

The catalytically active species of the enzyme (**A** in Figure 3) has a hydroxide ion coordinated to the Zn(II), in addition to the protein ligands (which in Figure 3 are those from LpCA1, *i.e.*, Cys90, His143 and Cys146). This is valid for all CAs belonging to the six different genetic families mentioned in the introduction. In the case of the α-CAs, the zinc hydroxide species is generated through a proton transfer process from the zinc coordinated water to the environment, assisted by the active site residue His64, acting as a proton shuttle [18]. In the β-CAs the activation of the zinc-coordinated water molecule (shown in **D** in Figure 3) is more complex than for the α-CAs, as the catalytic dyad mentioned earlier (Asp-Arg) takes part in the process. Furthermore, the nature of the amino acid(s) acting as proton shuttles in the β-class enzymes is not completely elucidated [23]. After the nucleophilic attack of the zinc hydroxide species of the enzyme on the bound CO_2_ (shown in **B**) bicarbonate is formed (**C** in Figure 3) which might be bidentately coordinated to the zinc ion (as in the α-CAs) [59]. Inorganic anions are usually weak ligands of the zinc ion in metalloenzymes, so that species **C** is probably easily converted to species **D**, by a substitution reaction of the bicarbonate by means of a water molecule. This leads to liberation of the reaction product (bicarbonate) into solution, with generation of the acidic form of the enzyme, **D**, which is catalytically inactive. To generate the zinc hydroxide, catalytically effective species **A**, a proton transfer reaction must occur (Figure 3), probably assisted by an active site residue. In the β-CA from the alga *Coccomyxa*, it has been hypothesized that there are two residues participating in this process, Tyr88 and His92 [60]. However, neither LpCA1 nor LpCA2 possess a sequence of Tyr and His residues close to each other as the algal enzyme, which leads the nature of the proton shuttling residue in these novel β-CAs unknown at this moment. The inhibition mechanism of LpCA1 and LpCA2 (shown in steps **E** and **F** in Figure 3) involves the inhibitor substituting the fourth, non-protein zinc ligand, leading to tetrahedral species, as those shown in **E**. Anions such as acetate bound to Can2 [50], as well as thiocyanate, azide, and phosphate bound to the *Coccomyxa* β-CA [60], were shown to possess this inhibition mechanism by means of high resolution X-ray crystallography. An alternative inhibition mechanism has been also reported again for the *Coccomyxa* β-CA for iodide [60], which was found anchored to the zinc-coordinated water molecule/hydroxide ion, as shown schematically in Figure 3, **F**. This mechanism was observed for phenols [61], polyamines [62], and sulfocoumarins [63] for the inhibition of α-CAs and may be more general than originally considered [23].

## 5. Conclusions

*Legionella pneumophila* is a Gram-negative bacterium causing Legionnaires' disease and infections caused by it are increasing all over the world. Two β-CAs were recently identified, cloned and purified in this pathogen (LpCA1 and LpCA2) [43,44] and considering the successful inhibition of growth of other bacteria when their CA was inhibited [57,58], the two enzymes were proposed as possible drug targets. LpCA1/LpCA2 efficiently catalyzed CO_2_ hydration to bicarbonate and protons, with k_cat_ in the range of (3.4–8.3) × 10^5^ s^−1^ and k_cat_/K_M_ of (4.7–8.5) × 10^7^ M^−1^·s^−1^, being thus among the very efficient bacterial CAs discovered and characterized so far. They were inhibited by many sulfonamides and sulfamates, the main class of CAIs, with several nanomolar inhibitors detected, although few studies are available in the literature to date [44]. The best LpCA1 inhibitors were aminobenzolamide and structurally similar sulfonylated aromatic sulfonamides, as well as acetazolamide and ethoxzolamide (K_I_s in the range of 40.3–90.5 nM). The best LpCA2 inhibitors belonged to the same class of sulfonylated sulfonamides, together with acetazolamide, methazolamide, and dichlorophenamide (K_I_s in the range of 25.2–88.5 nM). Inorganic anions were millimolar inhibitors, whereas diethyldithiocarbamate, sulfamate, sulfamide, phenylboronic acid, and phenylarsonic acid were micromolar ones against both bacterial enzymes. As these enzymes may be involved in pH regulation in the phagosome during *Legionella* infection, their inhibition may lead to antibacterials with a novel mechanism of action, but *in vivo* studies are necessary in order to validate them definitively as drug targets.

## Figures and Tables

**Table 1:** Kinetic parameters for the CO_2_ hydration reaction catalyzed by the human cytosolic isozymes hCA I and II (α-class CAs [18,19,20,21,22]) at 20 °C and pH 7.5 in 10 mM HEPES buffer and 20 mM Na_2_SO_4_, and the β-CAs Can2, CalCA (from *C. neoformans* and *C. albicans*, respectively) [50,51], SceCA (from *S. cerevisiae*) [52], *H. pylori* β-CA (HpyCA) [45], *B. suis* (BsuCA219 and BsuCA213) [46,47] and the two *L. pneumophila* enzymes LpCA1 and LpCA2 [43,44], measured at 20 °C, pH 8.3. Inhibition data with the clinically used sulfonamide acetazolamide (5-acetamido-1,3,4-thiadiazole-2-sulfonamide, **AAZ**) are also shown.

Isozyme	Activity Level	Class (s^−1^)	k_cat_ (M^−1^·s^−1^)	k_cat_/K_m_ (nM)	K_I_ (AAZ)	Ref.
hCA I	moderate	α	2.0 × 10^5^	5.0 × 10^7^	250	[18]
hCA II	very high	α	1.4 × 10^6^	1.5 × 10^8^	12	[18]
Can2	moderate	β	3.9 × 10^5^	4.3 × 10^7^	10.5	[50]
CalCA	high	β	8.0 × 10^5^	9.7 × 10^7^	132	[51]
SceCA	high	β	9.4 × 10^5^	9.8 × 10^7^	82	[52]
HpyCA	moderate	β	7.1 × 10^5^	4.8 × 10^7^	40	[45]
BsuCA219	moderate	β	6.4 × 10^5^	3.9 × 10^7^	63	[46]
BsuCA213	high	β	1.1 × 10^6^	8.9 × 10^7^	303	[47]
LpCA1	moderate	β	3.4 × 10^5^	4.7 × 10^7^	76.8	[43,44]
LpCA2	high	β	8.3 × 10^5^	8.5 × 10^7^	72.1	[43,44]

**Table 2:** Domain, species, accession numbers, and cryptonyms of the β-CA amino acid sequences used to construct the phylogenetic tree of Figure 2.

Domain	Species	Accession Number	Cryptonym
Bacteria	*Legionella pneumophila*	WP_014844650.1	LpCA1
**	*Legionella pneumophila*	WP_014842179.1	LpCA2
**	*Myroides injenensis*	ZP_10784819.1	MinCA
**	*Porphyromonas gingivalis*	YP_001929649.1	PgiCA
**	*Acinetobacter baumannii*	YP_002326524	AbaCA
**	*Escherichia coli*	ACI70660	EcoCa
**	*Helicobacter pylori*	BAF34127.1	HpyCA
**	*Burkholderia thailandensis* Bt4	ZP_02386321.1	BthCA
**	*Brucella suis* 1330	NP_699962.1	BsuCA
Archaea	*Methanobacterium thermoautotrophicum*	GI:13786688	Cab
Eukaryota (fungus)	*Saccharomyces cerevisiae*	GAA26059	SceCA
**	*Dekkera bruxellensis AWRI1499*	EIF49256	DbrCA
**	*Schizosaccharomyces pombe*	CAA21790	SpoCA
Eukaryota (green alga)	*Coccomyxa* sp.	AAC33484.1	CspCA
**	*Chlamydomonas reinhardtii*	XP_001699151.1	CreCA
Eukaryota (green plant)	*Vigna radiata*	AAD27876	VraCA
**	*Flaveria bidentis*, isoform I	AAA86939.2	FbiCA
**	*Zea mays*	NP_001147846.1	ZmaCA
**	*Arabidopsis thaliana*	AAA50156	AthCA

**Table 3:** Inhibition of human isoforms hCA I and hCA II, and of the β-class bacterial enzymes from *H. pylori* (HypCA) and *L. pneumophila* (LpCA1 and LpCA2) with sulfonamides **1**–**24** and the clinically used drugs **AAZ—HCT** [44].

Inhibitor	K_I_ (nM)				
Enzyme Class	hCA I	hCA II	HpyCA	LpCA1	LpCA2
	α	α	β	β	β
**1**	28,000	300	nt	939	455
**2**	25,000	240	1845	946	277
**3**	79	8	nt	1060	933
**4**	78,500	320	2470	556	624
**5**	25,000	170	2360	757	516
**6**	21,000	160	3500	734	375
**7**	8300	60	1359	770	592
**8**	9800	110	1463	866	396
**9**	6500	40	1235	988	181
**10**	7300	54	nt	913	622
**11**	5800	63	973	929	593
**12**	8400	75	640	642	496
**13**	8600	60	2590	541	382
**14**	9300	19	768	913	391
**15**	5500	80	nt	969	280
**16**	9500	94	236	2260	631
**17**	21,000	125	218	3540	721
**18**	164	46	450	2390	476
**19**	109	33	38	472	321
**20**	6	2	64	90.5	45.1
**21**	69	11	nt	101	78.9
**22**	164	46	nt	319	52.3
**23**	109	33	87	59.8	50.1
**24**	95	30	71	40.3	25.2
**AAZ**	250	12	40	76.8	72.1
**MZA**	50	14	176	201	88.5
**EZA**	25	8	33	71.4	103
**DCP**	1200	38	105	1670	64.1
**DZA**	50,000	9	73	2070	336
**BRZ**	45,000	3	128	648	467
**BZA**	15	9	54	159	148
**TPM**	250	10	32	665	882
**ZNS**	56	35	254	831	820
**SLP**	1200	40	35	253	245
**IND**	31	15	143	1090	525
**VLX**	54,000	43	nt	536	879
**CLX**	50,000	21	nt	990	421
**SLT**	374	9	nt	485	463
**SAC**	18,540	5959	nt	20,500	441
**HCT**	328	290	nt	15,800	745

nt = not tested.

**Table 4:** Inhibition constants of anionic inhibitors against α-CA isozymes derived from human (hCA II) and the β-CAs from bacteria (*H. pylori*, HpyCA), and the two enzymes LpCA1 and LpCA2 from *L. pneumophila* [43].

Inhibitor ^§^	K_I_ (mM)			
	hCA II	HpyCA	LpCA1	LpCA2
	α	β	β	β
F^−^	>300	0.67	0.91	0.77
Cl^−^	200	0.56	0.79	0.81
Br^−^	63	0.38	0.65	8.0
I^−^	26	0.63	0.32	59.1
CNO^−^	0.03	0.37	0.66	0.96
SCN^−^	1.60	0.68	0.52	0.88
CN^−^	0.02	0.54	0.064	0.61
N_3_^−^	1.51	0.80	0.077	0.45
HCO_3_^−^	85	0.50	3.5	6.6
CO_3_^2−^	73	0.42	4.7	4.8
NO_3_^−^	35	0.78	7.6	30.1
NO_2_^−^	63	0.67	7.9	5.8
HS^−^	0.04	0.58	0.076	0.51
HSO_3_^−^	89	0.63	6.6	7.2
SnO_3_^2−^	0.83	0.48	0.57	0.63
SeO_4_^2−^	112	0.65	7.3	0.66
TeO_4_^2−^	0.92	0.45	0.24	0.29
P_2_O_7_^4−^	48.50	0.75	0.94	0.83
V_2_O_7_^4−^	0.57	0.18	0.39	0.47
B_4_O_7_^2−^	0.95	0.68	0.60	0.55
ReO_4_^−^	0.75	0.82	0.89	0.77
RuO_4_^−^	0.69	1.10	0.82	0.86
S_2_O_8_^2−^	0.084	0.93	0.85	0.57
SeCN^−^	0.086	0.97	0.98	0.66
CS_3_^2−^	0.0088	0.21	0.53	0.62
Et_2_NCS_2_^−^	3.1	0.0074	0.006	0.002
SO_4_^2−^	>200	0.57	77.9	96.5
ClO_4_^−^	>200	6.50	>200	>200
BF_4_^−^	>200	>200	>200	>200
FSO_3_^−^	0.46	0.75	9.1	0.46
NH(SO_3_)_2_^2−^	0.76	0.70	1.17	0.59
H_2_NSO_2_NH_2_	1.13	0.072	0.094	0.009
H_2_NSO_3_H	0.39	0.094	0.076	0.013
Ph-B(OH)_2_	23.1	0.073	0.065	0.006
Ph-AsO_3_H_2_	49.2	0.092	0.084	0.008

^§^ As sodium salts.
